# Enlargement of the Spleen, of Nineteen Years’ Standing, Cured with Iodine in Five Months

**Published:** 1868-03

**Authors:** A. Given

**Affiliations:** Louisville, Ky.


### ENLARGEMENT OF THE SPLEEN, OF NINETEEN YEARS' STANDING, CURED WITH IODINE IN FIVE MONTHS.

By A. GIVEN, M.D., Louisville, Ky.

I was consulted, August 15th, 1867, by Mrs. Hinkle, on account of a tumor in the left side of the abdomen. She stated that 20 years previous, when 9 years of age, she had chills, which lasted for 9 months. About that time, there was a fulness observed at the margin of the ribs, on the left side, which her physician said was caused by an enlargement of the spleen, and for which she was treated by several physicians without any relief.

She is the mother of four children, and in the enjoyment of very good health. The tumor had never given her any inconvenience until after the birth of her youngest child, two years ago; since that time it had increased very rapidly. At the time I examined her, there was no pain or tenderness, and the only inconvenience she felt was from its weight, when attending to her household duties. The abdominal parietes being lax and pretty free from adipose, presented a favorable opportunity for diagnosing.

Upon examination, I found the following characteristics of the tumor: its upper border lying below the margin of the ribs, attached to a cord-like substance. I traced it around to the left into the lumbar region, down to the crest of the ilium; lying close to that bone, I traced it around to Poupart's ligament, and along the pubis to a little beyond the median line; thence, in a slightly irregular line, to the umbilicus; thence, in a curved line, to the cord-like substance before spoken of. The iliac and pubic borders were thin and irregular, corresponding with the irregularity of those parts. The umbilical border was round, and had a ridge-like feel; the surface was slightly concave or slightly depressed between the thick umbilical border and the fluting iliac border.

In order to get a correct diagnosis, I placed the patient in the recumbent position, elevated her hips, and began to knead the lower part of the tumor; it readily passed upwards and outwards, until it caused difficulty of breathing by its pressure against the diaphragm.

During the examination, the question arose in my mind, whether the tumor was malignant, ovarian, or enlargement of the spleen. The first was readily dismissed, on account of the general health, freedom from pain, and the complexion of the patient. But it was not so easy to diagnosticate between ovarian tumor and an enlarged spleen, inasmuch as the largest portion of the tumor occupied the usual position of ovarian. The irregularity of the tumor and its mobility beyond the usual situation of ovarian tumors, enabled me to pronounce it a case of enlargement of the spleen. After consulting Wood, and some notes which I had taken some years ago, I felt no doubt as to the correctness of my diagnosis. During the winter of 1858, while attending the clinics of Dr. N. S. Davis, in the medical wards of Mercy Hospital, Chicago, a case was presented to the class for examination, on the 28th of October, which was similar to the one now under consideration, except the tumor was accompanied with a dropsical effusion of the abdomen, which rendered it very difficult to diagnosticate between ovarian tumor and an enlarged spleen. I was not present when the case was again brought before the class, and cannot say whether the woman recovered or not; but the remarks of Professor Davis made an impression on my mind, as to the possibility of the spleen becoming so much distended; and he gave such a fine description of the pathology of those cases and the method of diagnosing them, which did very much to clear up the doubts from my mind as to the character of the present case. Then, again, while I had charge of the Refugee Hospital, at Louisville, in January, 1865, I admitted a girl, 12 years of age, to the medical ward, who was in the last stage of phthisis. I learned from her mother that about a year previous she had had intermittent fever, which lasted her for several months, leaving her with a cough and enlargement of the left side, near the margin of the ribs. On examination, I found a large wedge-like tumor lying loose in the left side of the abdominal cavity against the pubis. She died in a few days after being admitted. I made a *post mortem* and found the tumor to be the spleen lying entirely from under the ribs and against the pubis.

Being satisfied as to my diagnosis and the character of the tumor, the next point of interest was as to the treatment to be adopted. I could not see much prospect of success from the ordinary treatment, as laid down for splenitis. It was evident that if relieved at all, it must be by resolvents and absorbents. With this therapeutic indication in view, I put her upon the following:

℞. Iodine,------------------------------ gr. x.

Iodide Potassa,--------------------- ℨij.

Water,------------------------------ ℥j.

M., and give 12 drops, largely diluted with sweetened water, after each meal; and ordered the following liniment to be used:

℞. Camphor Soap Liniment, ---------------- ℥vj.

Tr. Iodine, -------------------------------- ℥ij.

M., and apply to the tumor twice a-day.

I gave directions that if the stomach became irritable, to discontinue the treatment for a few days. After following the above treatment for two months, I again examined the tumor, and found it softer and diminished one-half. I continued the treatment, with the same precautions as to the effects of the medicine on the system. I made another examination on the 15th of January, 1868, and found the tumor entirely gone, except a little fulness near the margin of the ribs.

I confess that I had no hopes of such a favorable result at my first examination; for it is seldom that we can get a patient to continue our treatment long enough to remove those large morbid growths, and to overcome the pathological condition necessary to their production and propagation. But by the rapid increase of the tumor, she was easily convinced that unless something was done soon, serious results would follow.

